# Does Full-Mouth Disinfection Influence the Size of the Periodontal Inflammatory Burden and the Level of hsCRP?

**DOI:** 10.3290/j.ohpd.b5245853

**Published:** 2024-04-23

**Authors:** Eva Skaleric, Nina Hropot Plesko

**Affiliations:** a Assistant Professor and Periodontist, Department of Oral Diseases and Periodontology, University Medical Centre and Medical Faculty, Ljubljana, Slovenia. Idea, experimental design, performed the treatment, wrote the manuscript.; b Teaching Assistant and Periodontist, Department of Oral Diseases and Periodontology, University Medical Centre and Medical Faculty, Ljubljana, Slovenia. Performed the treatment, wrote the manuscript.

**Keywords:** C-reactive protein, full-mouth disinfection, periodontal inflammatory burden, periodontal wound

## Abstract

**Purpose:**

To investigate the effect of full-mouth disinfection on the sizes of the periodontal wound and periodontal inflammatory burden and whether it leads to a decrease in C-reactive protein (CRP) levels.

**Materials and Methods:**

The study included 20 systemically healthy subjects (11 women and 9 men) 30 to 68 years old with localised or generalised periodontitis (stage III, grade C). The sizes of the periodontal wound and periodontal inflammatory burden were measured with the web application “Periodontalwound”, which is based on measurements of average tooth cervices, as well as probing depths and bleeding on probing assessed at six sites around each tooth present in the oral cavity. The levels of hsCRP (high-sensitivity CRP) were measured with an immunochemical method. All three parameters were measured before initial treatment and 3 months after therapy. Full-mouth disinfection included removal of plaque and calculus with ultrasonic and hand instruments in one session.

**Results:**

The results showed a statistically significant decrease in the size of the periodontal wound (p < 0.001), a statistically significant decrease in the size of periodontal inflammatory burden (p < 0.001), and a decrease in hsCRP levels 3 months after therapy.

**Conclusion:**

Full-mouth disinfection leads to a decrease in the periodontal wound and periodontal inflammatory burden size, as well as a decrease in the levels of hsCRP in patients with localised or generalised periodontitis (stage III, grade C).

Periodontal disease is a highly prevalent chronic inflammatory disease characterised by the destruction of supportive tissues of the affected teeth.^11,30^ Despite reports on a decrease in the prevalence of periodontitis in many studies,^17^ the most recent study on Ljubljana citizens showed that the prevalence of periodontal disease among the adult population of Ljubljana is still high. All Ljubljana citizens between the ages of 45 and 95 need oral hygiene instruction, 96.6% also need root planing and scaling, and 47.7% additionally need complex periodontal treatment.^36^ Observational studies have shown that untreated periodontal disease not only leads to tooth loss but also represents an increased risk for several systemic complications,^8,15^ such as cardiovascular events,^9,33^ metabolic syndrome,^31^ diabetes,^7,24^ adverse pregnancy outcomes,^39^ Alzheimer disease^21^ and intestinal inflammation.^22^ In periodontitis, bacteria and their products enter into the bloodstream during tooth extraction, scaling and root planing, as well as toothbrushing and mastication.^38^

Periodontal pockets are subgingival, inflamed and ulcerated areas which are subject to mechanical forces most of the time. Procedures such as mastication, toothbrushing and professional cleaning lead to transient bacteraemia, which is correlated to gingival inflammation.^38^ Cells within the connective tissue underlying periodontal pockets may also secrete inflammatory mediators.^3^ Nesse et al^26^ quantified the periodontal inflamed surface area to be from 0.3 cm^2^ in healthy people to 39 cm^2^ in patients with severe periodontitis. Later, Skaleric et al^38^ developed a similar method with which they calculated the periodontal inflammatory burden area in 238 random 35- to 85-year-old subjects. The inflammatory burden was an average of 9.25 ± 5.57cm^2^. The inflammatory burden area was also statistically significantly (p < 0.05) correlated with increased levels of C-reactive protein (CRP).

To estimate the size of the total area that is ulcerated and inflamed in periodontitis patients, a method for evaluating periodontal wound and periodontal inflammatory burden was developed. To evaluate these areas, measurements of average tooth cervices, probing depths (PD) and information about the presence of bleeding on probing (BOP) at six sites around each tooth are needed.^38^ On the basis of this method, a web application (web app), www.periodontalwound.info (Periodontalwound, Adal d.o.o. and Skaldens; Ljubljana, Slovenia) was developed. The web app Periodontalwound allows this method to be used clinically. With this app, the sizes of the periodontal wound and periodontal inflammatory burden can be calculated chairside. The periodontal wound is a subgingival area that includes all sites that bleed on probing within the oral cavity of an individual. Periodontal inflammatory burden represents all sites that bleed on probing in addition to all other sites that are inflamed but do not bleed on probing, as it is known that cells within the connective tissue underlying the periodontal pockets may secrete inflammatory mediators such as CRP, plasminogen activator^1^ and fibrinogen,^4^ which in turn may lead to systemic problems.^3^ By using the web app Periodontalwound, the clinician can show the patient the extent of the wound and inflamed area, representing periodontal disease, in their oral cavity (Fig 1). This can also be used as a tool for showing the patient that periodontal disease affects their systemic health. After effective therapy, the patient can see the decrease in the sizes of the periodontal wound and periodontal inflammatory burden.

**Fig 1:**
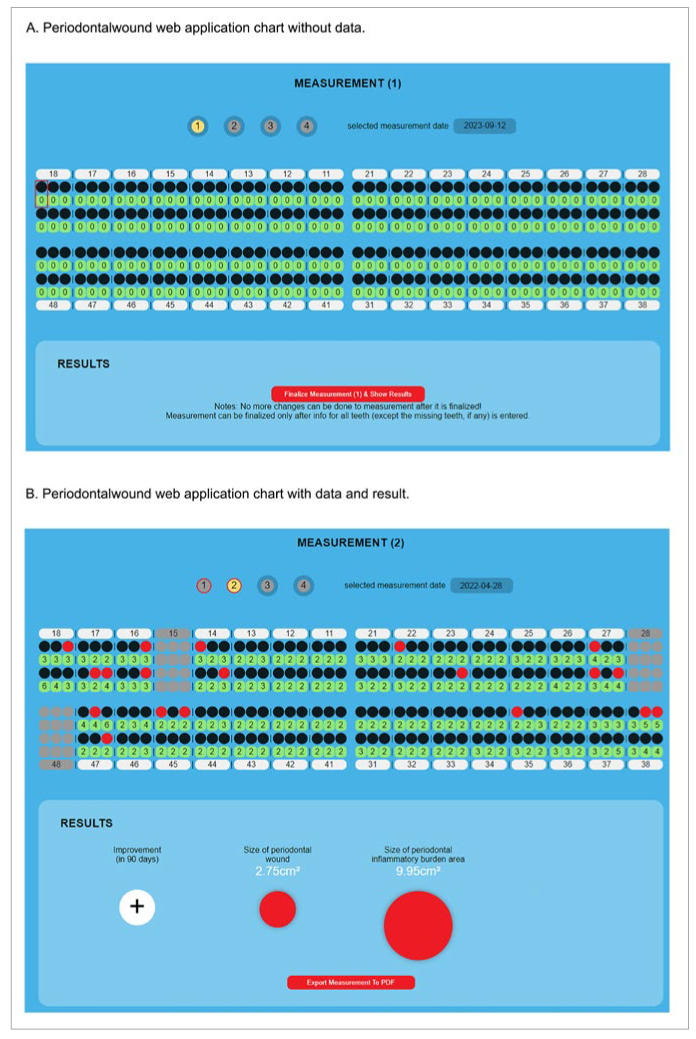
Example of the Periodontalwound web app without (A) and with entered data (B).

One of the possible explanations for the causal association between periodontal disease and cardiovascular diseases is an increased serum CRP level in patients with periodontal disease.^35^ Many studies have shown that elevated CRP levels can lead to cardiovascular disease. A meta-analysis of studies reported that CRP levels in patients with periodontal disease are 1.56 mg/l higher comparing to patients without periodontal disease,^29^ which can be clinically relevant.^16^ One of the studies^38^ showed that hsCRP (high-sensitivity-CRP) levels increase with periodontal inflammatory burden in systemically healthy individuals. A hsCRP test can detect very low values of CRP (0.1 mg/l) in the serum. People with hsCRP values < 1 mg/l have a low risk, people with hsCRP values 1–3 mg/l have a moderate risk and people with hsCRP values > 3 mg/l have a high risk for cardiovascular diseases.^12^

Several studies have shown that non-surgical therapy leads to statistically significant improvements in clinical and microbiological parameters in periodontal patients.^1,2^ Quirynen et al^34^ explained the benefit of one-stage full-mouth disinfection (FMD). They indicated that the benefit was partially due to the use of antiseptics and partially due to the completion of therapy in one stage. However, Pockpa et al^32^ demonstrated that the results obtained with full-mouth disinfection and the conventional quadrant method are equivalent, and depend on the preferences of practitioner and the patient.

It has also been shown that non-surgical periodontal therapy statistically significantly reduces the level of serum CRP.^14,26^ Meta-analyses of studies also confirmed that periodontal treatment leads to a decrease in CRP levels.^28^

The aim of this study was to investigate whether FMD leads to a decrease in the sizes of the periodontal wound and periodontal inflammatory burden as well as a decrease in the levels of serum hsCRP. Our hypothesis is that FMD leads to a decrease of all three parameters.

## Materials and Methods

This interventional clinical study was conducted in accordance with the ethical principles of the Helsinki Declaration (WMA, 2013), and written informed consent was obtained. The study protocol was approved by the National Medical Ethics Committee of the Republic of Slovenia (No. 49/08/11).

All subjects were selected from the patients that were referred to the Department of Oral Diseases and Periodontology in the University Clinical Centre, Ljubljana, Slovenia. Twenty subjects (11 women and 9 men) 30 to 68 years old with localised or generalised periodontitis (stage III, grade C) fulfilled the inclusion criteria and were finally included in our study. The patients were systemically healthy, did not take any medication and had never smoked. They also had not taken any systemic antibiotics 3 months prior to the beginning of treatment. The subjects had to have at least 20 teeth.

For estimation of the periodontal wound and periodontal inflammatory burden, we used the web app Periodontalwound (www.periodontalwound.info), which can be used chairside. This application is based on the method for evaluation of the periodontal wound and periodontal inflammatory burden described previously by Skaleric et al.^37,38^ The method is based on measurements of average tooth cervices in addition to probing depths and bleeding on probing assessed at six sites around each tooth present in the oral cavity.

PD and BOP were assessed with a Williams’ periodontal probe (Hu Friedy; Chicago, IL, USA) at six sites around all teeth (disto-buccal, buccal, mesio-buccal, disto-lingual, lingual and mesio-lingual) present in the subject’s oral cavity. All data were entered chairside into the periodontal chart in the web app Periodontalwound. To obtain additional data, recession was measured at six sites around all teeth and clinical attachment level (CAL) was calculated.

To investigate the level of serum hsCRP, blood samples from fingertips of all subjects were collected before and 3 months after therapy. The level of hsCRP was then measured at the Clinical Institute for Clinical Chemistry and Biochemistry, University Medical Centre, Ljubljana, Slovenia. An immunochemical method with chemiluminescence detection on an IMMULITE automated analyser was used. This method detects even very low concentrations of hsCRP.

FMD included removal of plaque and calculus with ultrasonic and hand instruments in one session. Scaling and root planing was performed at sites with PD ≥ 4 mm. Subgingival instrumentation was performed under local anaesthesia. The tongue was then brushed with a 0.5% chlorhexidine gel (PERIOPLUS+ FOCUS, Curaprox, Curaden; Stutensee, Germany) for 1 min and the mouth was rinsed with 0.2% chlorhexidine solution (PERIOPLUS+ FORTE, Curaprox, Curaden) for 2 min. All pockets were rinsed with 0.2% chlorhexidine. After therapy, patients were advised to rinse with 0.2% chlorhexidine twice a day for two weeks. All patients received oral hygiene instructions and motivation two weeks before FMD. Full-mouth plaque score (FMPS) was assessed prior to the beginning of therapy and was not allowed to exceed 20%.

All parameters were assessed prior to and at 3 months after therapy.

### Statistical Methods

The data were analysed using Stata 16 Statistical Package (StataCorp; College Station, TX, USA). Excel was used to generate the figures. Means and standard deviations were calculated for all parameters (hsCRP, periodontal wound size, periodontal inflammatory burden size, PD, BOP, CAL) before and after therapy. Mean values of each parameter before and after therapy were compared using the paired-sample t-test. A p-value < 0.05 was considered statistically significant, and p < 0.001 was considered highly statistically significant. Post-hoc power analysis of the study was calculated to be 99.77%.

## Results

### Patient Population

We included 20 systemically healthy subjects (11 women and 9 men) with localised or generalised periodontitis (stage III, grade C) in our study. They were 30 to 68 years old (mean age: 48). All patients completed the study. All patients had satisfactory plaque control (FMPS ≤ 20%) prior to FMD. Patients had from 23 to 32 teeth (mean: 24 teeth) in their oral cavity.

### Periodontal Wound and Periodontal Inflammatory Burden

**Fig 2:**
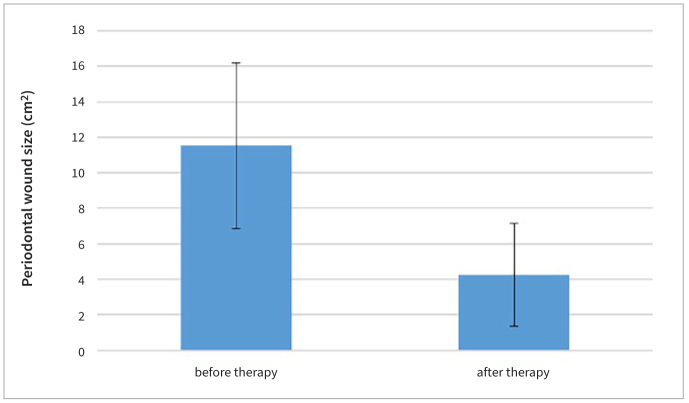
Mean periodontal wound size before and after FMD.

**Fig 3:**
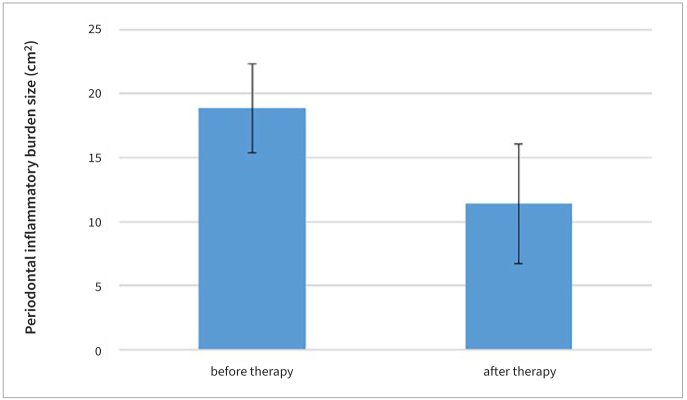
Periodontal inflammatory burden size before and after FMD.

**Fig 4:**
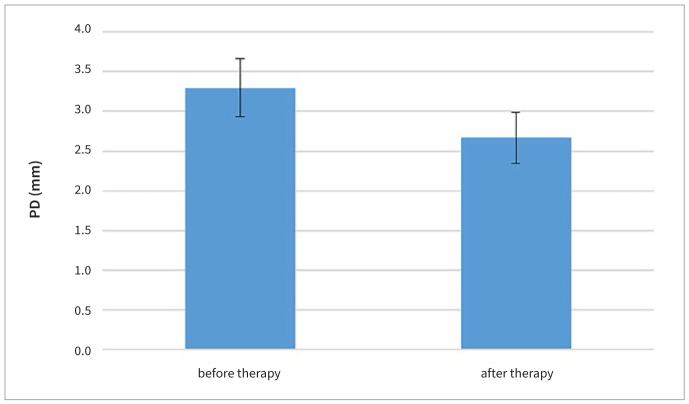
Mean probing depth before and after FMD.

**Fig 5:**
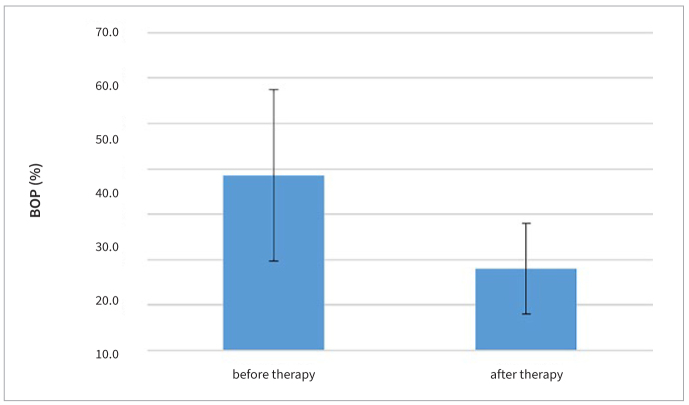
The percentage of sites with BOP before and after FMD.

Both the sizes of the periodontal wound and periodontal inflammatory burden decreased 3 months after FMD. The average size of the periodontal wound was 11.53 cm^2^ ± 4.66 prior to FMD and 4.26 cm^2^ ± 2.91 3 months after FMD, an average size decrease (Fig 2) of 7.27 cm^2^ (p < 0.001) (Table 1). The average size of periodontal inflammatory burden was 18.85 cm^2^ ± 3.49 prior to FMD and 11.40 cm^2^ ± 4.69 3 months after FMD, an average decrease in size (Fig 3) of 7.45 cm^2^ (p < 0.001) (Table 2). Mean PD decreased (Fig 4) from 3.36 ± 0.26 mm to 2.85 ± 0.27 mm (p < 0.001) (Table 3) and mean BOP decreased (Fig 5) from 37% to 20% (p < 0.001) (Table 4). The average CAL was 3.23 ± 0.39 mm before FMD, and decreased to 2.63 mm ± 0.17 mm (p < 0.001) after 3 months.

**Table 1:** Comparison of mean periodontal wound size before and after FMD

Group	Mean ± SD (cm^2^)	t-value	p-value
Periodontal wound size (pre-treatment)	11.53 ± 4.47	6.311	<0.001
Periodontal wound size (post-treatment)	4.26 ± 2.91		

**Table 2:** Comparison of mean periodontal inflammatory burden size before and after FMD

Group	Mean ± SD (cm^2^)	t-value	p-value
Periodontal inflammatory burden size (pre-treatment)	18.85 ± 3.49	7.698	<0.001
Periodontal inflammatory burden size (post-treatment)	11.40 ± 4.69		

**Table 3:** Comparison of mean probing depth before and after FMD

Group	Mean ± SD (mm)	t-value	p-value
PD (pre-treatment)	3.36 ± 0.26	8.17	<0.001
PD (post-treatment)	2.85 ± 0.27		

PD: probing depth.

**Table 4:** Comparison of mean BOP before and after FMD

Group	Mean ± SD (%)	t-value	p-value
BOP (pre-treatment)	37.0 ± 11.0	4.95	<0.001
BOP (post-treatment)	20.0 ± 10.0		

BOP: bleeding on probing.

### Serum hsCRP Levels

**Fig 6:**
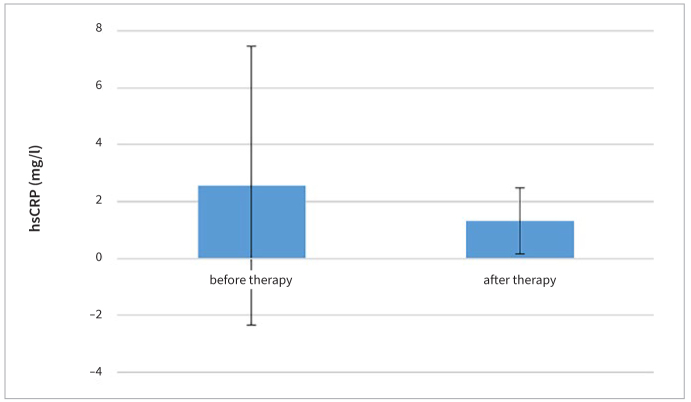
hsCRP level before and after FMD.

The baseline hsCRP was 2.55 ± 4.90 mg/l. Three months after FMD the hsCRP levels were 1.31 ± 1.15 mg/l; that is, they decreased (Fig 6) by 1.24 mg/l on average (p > 0.05) (Table 5).

**Table 5:** Comparison of mean hsCRP level before and after FMD

Group	Mean ± SD (mg/l)	t-value	p-value
hsCRP (pre-treatment)	2.55 ± 4.90	1.117	0.28
hsCRP (post-treatment)	1.31 ± 1.15		

hsCRP: high-sensitivity C-reactive protein.

## Discussion

The present study evaluated the effect of FMD on the sizes of periodontal wound, periodontal inflammatory burden, and the levels of serum CRP. Systemically healthy subjects with localised or generalised periodontitis (stage III, grade C) were included in the study in order to increase the accuracy of CRP-level measurment and systemic burden caused by periodontal disease.

The results showed that FMD led to an improvement of all periodontal parameters assessed, which is in accordance with many other studies.^10,20,23,32,34^ It was first thought that FMD was beneficial in comparison to quadrant-wise scaling, due to the use of antiseptics and the completion of therapy in one stage.^34^ However, many later studies^10,20,23,32^ showed that there are no statistically significant differences between the two techniques. Pockpa et al^32^ reported that full-mouth disinfection and the conventional quadrant method are equally effective, and depend on the preferences of the practitioner and the patient. Based on a systematic review and meta-analysis of multiple studies, Fang et al^13^ concluded that FMD has modest clinical benefits over Q-SRP and recommended FMD as the first choice for treatment of adult chronic periodontitis.

The present study investigated the effect of FMD on the sizes of periodontal wound and periodontal inflammatory burden. Skaleric et al^37,38^ presented a method for evaluation of periodontal wound and periodontal inflammatory burden to estimate the size of the total ulcerated and inflamed area in periodontitis patients. To evaluate this area, measurements of average tooth cervices, probing depths, and information about the presence of bleeding on probing at six sites around each tooth are needed. A web app, Periodontalwound (www.periodontalwound.info), was later developed to enable the method to be used clinically. With this app, the sizes of the periodontal wound and periodontal inflammatory burden can be calculated chairside by entering the probing depths and BOP at six sites around each tooth present in the oral cavity in the app chart. In the app, the average tooth cervix values and mathematical algorithms for calculation of both areas are included.

In this study, FMD led to a statistically significant decrease in the average size of the periodontal wound from 11.53 ± 4.66 cm^2^ to 4.26 ± 2.91 cm^2^ after 3 months. It also led to a statistically significant decrease of average periodontal inflammatory burden from 18.85 ± 3.49 cm^2^ to 11.40 ± 4.69 cm^2^ after 3 months.

Nesse et al^26^ developed a method for quantifying the periodontal inflamed surface area (PISA) in order to quantify inflammatory burden of periodontitis on systemic health. PISA is calculated using conventional clinical parameters such as BOP, combined with either PD or CAL, and gingival recession. The PISA was calculated to range from 0.3 cm^2^ in healthy individuals to 39 cm^2^ in patients with generalised periodontitis. These numbers are not in accordance with the results obtained in the present study, as our subjects had a diagnosis of localised or generalised periodontitis (stage III, grade C) and had an average periodontal inflammatory burden of 18.85 ± 3.49 cm^2^ prior to therapy, which is much lower. However, the present results are in accordance with the results of Hujoel et al,^18^ who reported a typical dentogingival epithelial surface area in patients with periodontitis as 8 to 20 cm^2^.

Nomura et al^27^ assessed the prospective longitudinal changes in the periodontal inflamed surface area following active periodontal treatment for chronic periodontitis. They included 125 patients with 3107 teeth in their study, and PISA was evaluated 5 times. For most patients, changes of PISA in the 24-month period after periodontal treatment were within 10% of the baseline. However, a number of bleeding sites at a tooth with a deep periodontal pocket was associated with an exponential increase in the PISA. It is difficult to compare the results of Nomura et al^27^ with our study, as a different model for assessing the periodontal inflamed area was used. In addition, Nomura et al^27^ did not include data on initial mean PISA values, so the values obtained in the two studies cannot be compared.

The results of our study showed that FMD led to a decrease in CRP serum levels. The baseline CRP was 2.55 ± 4.90 mg/l; three months after FMD, the CRP levels decreased to 1.31 ± 1.15 mg/l. These results are in accordance with results obtained by many other authors. D’Aiuto et al^6^ assessed serum CRP and IL-6 levels at baseline and 2 and 6 months after non-surgical periodontal therapy in 94 patients. Statistically significant reductions in both markers were found after therapy. Matilla et al^25^ also showed that the levels of hsCRP decreased after periodontal therapy. In a study by Cortelli et al,^5^ FMD was found to decrease CRP levels of both obese and non-obese individuals. However, some studies did not find statistically significant reductions of CRP levels after periodontal therapy.^19,40^

Our study is the first to investigate the effect of FMD on the sizes of the periodontal wound and periodontal inflammatory burden. It is also one of the few to assess the effect of FMD on the levels of serum CRP. By including only systemically healthy subjects, the impact of periodontal disease on CRP levels and consequently the systemic burden should be estimated quite accurately.

Another advantage of this study is also the use of the web app Periodontalwound, which allows the clinician to show the patient the sizes of the periodontal wound and periodontal inflammatory burden before and after treatment. It was also explained to the subjects who participated in the study that by decreasing the sizes of the periodontal wound and periodontal inflammatory burden, the possible impact of periodontal disease on their systemic health is lower.

One of the disadvantages of our study is that the web app Periodontalwound does not assess gingival recession. This means that in cases of gingival recession, the sizes of the periodontal wound and periodontal inflammatory burden are overestimated, and in cases of gingival hyperplasia, the sizes of the periodontal wound and periodontal inflammatory burden are underestimated. However, it is important to bear in mind that gingival recession does not contribute significantly to the impact on systemic health caused by periodontal disease.

## Conclusion

Full-mouth disinfection leads to a decrease in the sizes of periodontal wound and periodontal inflammatory burden and a decrease in the levels of CRP in patients with localised or generalised periodontitis (stage III, grade C). More interventional studies on a larger number of subjects are needed to prove that FMD leads to an improvement in the sizes of the periodontal wound and periodontal inflammatory burden and a decrease in serum CRP levels.
